# Evaluation of Circulating Cathodic Antigen (CCA) Urine-Tests for Diagnosis of *Schistosoma mansoni* Infection in Cameroon

**DOI:** 10.1371/journal.pntd.0001758

**Published:** 2012-07-31

**Authors:** Louis-Albert Tchuem Tchuenté, Césaire Joris Kueté Fouodo, Romuald Isaka Kamwa Ngassam, Laurentine Sumo, Calvine Dongmo Noumedem, Christian Mérimé Kenfack, Nestor Feussom Gipwe, Esther Dankoni Nana, J. Russell Stothard, David Rollinson

**Affiliations:** 1 Centre for Schistosomiasis and Parasitology, Yaoundé, Cameroon; 2 Laboratory of Parasitology and Ecology, Faculty of Sciences, University of Yaoundé I, Yaoundé, Cameroon; 3 National Programme for the Control of Schistosomiasis and Intestinal Helminthiasis, Ministry of Public Health, Yaoundé, Cameroon; 4 Université de Yaoundé, IRD UMI 209 UMMISCO, Yaoundé, Cameroon; 5 Disease Control Strategy Group, Liverpool School of Tropical Medicine, Liverpool, United Kingdom; 6 Wolfson Wellcome Biomedical Laboratories, The Natural History Museum, London, United Kingdom; Centers for Disease Control and Prevention, United States of America

## Abstract

**Background:**

The Kato-Katz is the most common diagnostic method for *Schistosoma mansoni* infection. However, the day-to-day variability in host egg-excretion and its low detection sensitivity are major limits for its use in low transmission zones and after widespread chemotherapy. We evaluated the accuracy of circulating cathodic antigen (CCA) urine-assay as a diagnostic tool of *S. mansoni*. In comparison, a low sensitive CCA test (CCA-L) was assessed.

**Methodology:**

The study was conducted in three settings: two foci with single *S. mansoni* infections (settings A and B), and one mixed *S. mansoni – S. haematobium* focus (setting C). Stool and urine samples were collected from school-children on three consecutive days. Triplicate Kato-Katz readings were performed per stool sample. Each urine sample was tested with one CCA and only the first urine sample was subjected to CCA-L. Urine samples were also examined for *S. haematobium* eggs using the filtration method and for microhaematuria using urine reagent strips. Overall, 625 children provided three stool and three urine samples.

**Principal Findings:**

Considering nine Kato-Katz thick smears as ‘reference’ diagnostic test, the prevalence of *S. mansoni* was 36.2%, 71.8% and 64.0% in settings A, B and C, respectively. The prevalence of *S. haematobium* in setting C was 12.0%. The sensitivities of single Kato-Katz, CCA and CCA-L from the first stool or urine samples were 58%, 82% and 46% in setting A, 56.8%, 82.4% and 68.8% in setting B, and 49.0%, 87.7% and 55.5% in setting C. The respective specificities were 100%, 64.7% and 100%; 100%, 62.3% and 91.3%; and 100%, 42.5% and 92.0%. Mixed infection with *S. haematobium* did not influence the CCA test results for *S. mansoni* diagnosis.

**Conclusions/Significance:**

Urine CCA revealed higher sensitivity than CCA-L and triplicate Kato-Katz, and produced similar prevalence as nine Kato-Katz. It seems an attractive method for *S. mansoni* diagnosis.

## Author Summary

Recent momentum for the control of neglected tropical diseases has generated a renewed interest in the control of schistosomiasis, resulting in the increase of large scale mass drug administration of praziquantel in several countries. As a consequence, there is a need to develop more sensitive diagnostic tools, especially for Schistosoma mansoni, in order to help targeting control or develop better screening strategies for elimination. We evaluated the commercially available point-of-contact circulating cathodic antigen (CCA) as a potential tool for the screening of *S. mansoni* infections. Our results showed that urine CCA assay had a better sensitivity for detecting S. mansoni infections than single and triplicate Kato-Katz thick smears. The prevalence obtained with CCA was similar or higher than that obtained with nine Kato-Katz. Neither STH nor *S. haematobium* infections had any impact on the CCA test results. Although further studies are required for its refinement and validation, the overall results revealed CCA as a promising and easy to use tool for the diagnosis of *S. mansoni*.

## Introduction

Schistosomiasis remains of significant public health importance worldwide, with an estimated 207 million people infected. Over 90% of all schistosomiasis cases are found in sub-Saharan Africa [1], [2]. The disease is caused by six species of blood flukes: *Schistosoma haematobium*, *S. mansoni*, *S. japonicum*, *S. intercalatum*, *S. guineensis* and *S. mekongi*. *S. haematobium* causes urogenital schistosomiasis while the other species cause intestinal disease. The disease affects the poorest of the poor and compromises their development [3]. The development of preventive chemotherapy strategy, the greater access to praziquantel and the increased resources for control have amplified treatment possibilities to the majority of people who need it [4]. Significant progress has been made in the control of schistosomiasis within the past ten years, though this falls short of the target set by World Health Assembly Resolution 54.19 adopted in 2001, which aimed to reach at least 75% of all school-aged children at risk of morbidity by 2010 [5].

In sub-Saharan Africa, *S. haematobium* and *S. mansoni* are the major causes of disease. *S. haematobium* infections can be diagnosed by several approaches, including detection of schistosome eggs in urines and rapid tests such as urine reagent strips for detection of microhaematuria [6]. On the contrary, there is currently no validated rapid test for *S. mansoni*. Because of its simplicity and relatively low-cost, the Kato-Katz technique [7] is widely used for epidemiological field surveys and is recommended by WHO for mapping to determine programme intervention zones, and for monitoring, evaluation and surveillance of intestinal schistosomiasis control programmes [8]. Though the specificity is very high, the sensitivity of Kato-Katz in single stool sample examination is limited by day-to-day variation in egg excretions leading to measurement error in estimating the presence of infection. This is particularly accentuated in areas with high proportions of light intensity infections [9]–[11]. The increase of large-scale interventions and repeated mass treatment with praziquantel will significantly reduce the prevalence and intensities of schistosomiasis. As consequence of the increase of low-intensity schistosome infections, more light infections will be often missed if single stool samples are examined by the Kato-Katz method, resulting in high underestimation of infection. Therefore, there is a need to develop and validate more sensitive diagnostic tools for *S. mansoni* infections.

Within the Schistosomiasis Consortium for Operational Research and Evaluation (SCORE) framework, a multi-country evaluation of the commercially available point-of-contact (POC) circulating cathodic antigen (CCA) was conducted. The study aimed to evaluate the utility of the POC urine-based CCA assay as a survey tool to determine the prevalence of *S. mansoni*. The sensitivity and specificity of the POC-CCA for diagnosis of *S. mansoni* were assessed, using the cumulative results of the 9 Kato-Katz thick smear readings as ‘reference’ diagnostic test. In Cameroon, our study was conducted in three distinct epidemiological settings at different endemic levels; two areas of low and moderate endemicity of *S. mansoni*, and one mixed infection focus of *S. mansoni* and *S. haematobium*. As previous data suggested that POC-CCA testing may result in more children believed to be positive than by Kato-Katz testing, with a portion of these being false positives, the manufacturer developed a less sensitive version of POC-CCA. Therefore, in order to further assess the performance of CCA test, this experimental low sensitive CCA dipstick (designated CCA-L) was also tested in comparison to the commercially available CCA assay (designated CCA).

## Methods

### Ethical statement

The study was approved by the National Ethics Committee of Cameroon (Nr 084/CNE/DNM/09), and was a public health exercise through the Ministry of Health and the Ministry of Education. Stool and urine samples were collected from children in schools with the approval of the administrative authorities, school inspectors, directors and teachers. The objectives of the study were explained to the schoolchildren and to their parents or guardians from whom written informed consent was obtained. Children willing to participate were registered. Each child was assigned an identification number and results were entered in a database and treated confidentially. No identification of any children can be revealed upon publication. All children who participated in the study were treated with praziquantel. Other children were treated during the MDA campaign implemented by the national programme for the control of schistosomiasis and intestinal helminthiasis.

### Study area

Based on previous parasitological data, three epidemiological settings were selected for the study: (i) one setting of low endemicity of single *S. mansoni* transmission, i.e. Yaoundé (setting A) in the Centre region of Cameroon; (ii) one setting of moderate endemicity of single S. mansoni transmission, i.e. Makenene (setting B), Centre region; and (iii) one setting where mixed infections of *S. mansoni* and *S. haematobium* occur, i.e. Njombe (setting C) in the Littoral region. Yaoundé is the capital city of Cameroon. Makenene and Njombe are located at approximately 200 and 325 km from Yaoundé, respectively. Investigations were conducted in public primary schools of Obobogo (3.82489 N, 11.50071 E) in Yaoundé, Baloua (4.88287 N, 10.78953 E) in Makenene, and Kompita (4.57898 N, 9.64848 E) in Njombe.

### Sampling and data collection

According to the literature, a single Kato-Katz thick smear for diagnosis of *S. mansoni* in low endemicity settings has a sensitivity of only 20–30% [10], [12]. However, since our study was to be carried out in both low and moderate endemicity settings, we assumed that a single Kato-Katz thick smear has a maximum sensitivity of 60%. The sensitivity of the CCA test is reported to be 80% or higher [13], [14]. Using these sensitivity estimates, a significance level of 5%, and a power of 80%, our sample size of complying children was calculated at 90. Assuming a compliance of 70% for the submission of each of three requested stool samples, the number of children to be included in each study setting was at least 199. To achieve this sample size, we selected a sample size of 250 children per study site.

The study was conducted between December 2010 and January 2011. In each of the three settings, about 250 schoolchildren from the upper classes (age 8–12 years) were enrolled, approximately half boys and half girls. Urine and stool samples were collected from these children over three consecutive days. The samples were collected between 11.00 and 14.00, in 60 mL plastic screw-cap vials, transported to the laboratory and processed the same day. In the laboratory, three Kato-Katz thick smear slides per stool sample, using 41.7 mg templates, were prepared and examined for *S. mansoni* and STH. All urine samples were tested using the CCA assays for diagnosis of *S. mansoni*. In addition, the first urine sample of each child was tested with CCA-L. Both CCA and CCA-L assays were obtained from Rapid Medical Diagnostics (Pretoria, South Africa) and performed at ambient temperature according to the manufacturer's instructions. Briefly, one drop of urine was added to the circular well of the test cassette and allowed to be absorbed entirely into the specimen pad within the well. Then, one drop of buffer (provided with the kit) was added. The test result was read 20 minutes after adding the buffer. Results were determined in a blinded fashion by at least two individuals. In case the control band did not develop, the test was considered as invalid. Valid tests were scored as negative, trace (weak band) or positive (strong band). Due to the lack of standards designed for this test, the trace and positive results were classified as positive.

In addition to the CCA tests, each urine sample was subjected to a filtration method for detection of *S. haematobium* eggs, and with reagent strips (Teco Diagnostics, USA) for microhaematuria assessment. Each urine sample was agitated to ensure adequate dispersal of eggs, 10 mL of urine were filtered through Nucleopore® filter, and filters were examined by microscopy for the presence of schistosome eggs. Schistosome infections were recorded; number of eggs was counted and intensity of infection was calculated and expressed as eggs per 10 mL of urine (eggs/10 mL) for *S. haematobium* or eggs per gram of feces (epg) for *S. mansoni*. For urinalysis, reagent strips were immersed in urine and removed immediately. The strip-results were read between 1–2 minutes by direct comparison of the colour blocks printed on the outside of the bottle label.

### Data analysis

The different data were analyzed by the epidemiological unit of the *Centre for Schistosomiasis & Parasitology* using appropriate statistical tests and methods. Data were entered in a Microsoft Excel spreadsheet, checked and validated. Statistical analyses were carried out using R software version 2.10.0. Only children with complete data records (i.e. 3 POC-CCA assays, 9 Kato-Katz thick smear readings, 3 reagent strip test results and 3 urine filtrations) were included in the final analysis. Sensitivity and specificity of CCA assays were estimated using 9 Kato-Katz thick smear readings as the reference test. The sensitivity was determined as the percentage of subjects with a positive CCA test among those positive for Kato-Katz; and the specificity was defined as the proportion of true negatives, i.e. the percentage of subjects negative for CCA among people negative for Kato-Katz. Positive predictive value (PPV) and negative predictive value (NPV) were also calculated for the different tests.

Analyses were performed for the 3 study settings separately to determine differences in the sensitivity of the CCA assays potentially resulting from different *S. mansoni* endemicity levels (infection prevalence and intensity) and as a function of *S. haematobium* co-infection. To obtain a standardized measure of infection, the geometric mean infection intensity of *S. mansoni*, expressed as the number of eggs per gram of stool (EPG), was estimated for the three study cohorts. For each individual, the classification into light (1–99 EPG), moderate (100–399 EPG) and heavy (≥400 EPG) infection intensity was calculated based on the arithmetic mean of EPGs derived from the 9 Kato-Katz thick smear readings. The thresholds are set by the World Health Organization [3]. The strength of agreement between the CCA and the 9 Kato–Katz thick smears for each endemic setting was assessed by kappa statistics (κ), as follows: k<0 indicating no agreement, k = 0–0.2 indicating poor agreement, k = 0.2–0.4 indicating fair agreement, k = 0.4–0.6 indicating moderate agreement, k = 0.6–0.8 indicating substantial agreement, and k = 0.8–1 indicating almost perfect agreement [15], [16]. Day-to-day variation of Kato-Katz results was assessed using McNemar test [17].

An ordinal logistic regression approach was performed to assess the correlation between CCA and CCA-L categories and *S. mansoni* egg counts. The geometric mean egg counts of nine Kato-Katz thick smears per stool sample per day served as continuous explanatory variable, whereas the color reaction of the CCA test was considered as categorical outcome. This statistical procedure was also used to assess the association between CCA and CCA-L test results, expressed as binary outcome variable (negative/positive), with *S. haematobium* egg count as continuous explanatory variable and micro-haematuria as categorical explanatory variable.

Non-overlapping 95% confidence intervals (CI) or p-values<0.05 were considered as statistical significance.

To further measure the discriminating power of the diagnostic CCA tests, receiver operating characteristic (ROC) curves were used to assess the association between sensitivity and specificity of the assays [18]. This allowed representing the variation of proportion of true positive individuals for Kato-Katz and CCA test in function of proportion of individuals negative for Kato-Katz but positive for CCA. The area under the curve (AUC) indicated the probability to identify accurately a true positive case when the result was simultaneously positive and negative for the CCA test, using single, triplicate and 9-Kato-Katz as the reference tests. AUC>0.7 indicates high discriminating power.

## Results

### Study adherence

A total of 765 pupils were registered and included in the study: 258 in setting A, 251 in setting B and 256 in setting C. Of these children registered, 625 (81.7%) aged 7–15 years old (mean 10.7 years) provided all three requested urine and stool samples: 138 (53.5%) in setting A, 245 (97.6%) in setting B and 242 (94.5%) in setting C.

### Prevalence of *S. mansoni* (Kato-Katz)

Table 1 summarizes the results of schistosomiasis prevalence obtained in the different study settings, as assessed by the different diagnostics approaches. The 95% confidence intervals (95% CI) for prevalence are shown. For the Kato-Katz method, there was a significant increase of infection prevalence with the increase of the number of Kato-Katz thick smear readings. Indeed, the prevalence figures for *S. mansoni* were 21.0%, 23.9% and 36.2% in setting A; 41.0%, 49.0% and 71.8% in setting B; and 31.4%, 44.6% and 64.0% in setting C; with one, three and nine Kato-Katz thick smears, respectively.

**Table 1:** Prevalence of *S. mansoni* and *S. haematobium* infections according to each diagnostic test and stratified by epidemiological setting.

	Setting A			Setting B			Setting C			All Settings A-B-C		
Diagnostic test	No. of children tested	No. of children positive	% positive (95% CI)	No. of children tested	No. of children positive	% positive (95% CI)	No. of children tested	No. of children positive	% positive (95% CI)	No. of children tested	No. of children positive	% positive (95% CI)
***S. mansoni*** ** diagnosis**												
Nine Kato-Katz	138	50	36.2	245	176	71.8	242	155	64.0	625	381	61.0
			(28.2–44.3)			(66.2–77.5)			(58.0–70.1)			(57.1–64.8)
Three Kato-Katz (stool day 1)	138	33	23.9	245	120	49.0	242	108	44.6	625	261	41.8
			(16.8–31.0)			(42.7–55.2)			(38.4–50.9)			(37.9–45.6)
One Kato-Katz (stool day 1)	138	29	21.0	245	100	41.0	242	76	31.4	625	205	32.8
			(14.2–27.8)			(34.7–47.0)			(25.6–37.3)			(29.1–36.5)
Three CCA	138	96	62.3	245	205	83.7	242	209	86.4	625	510	81.6
			(54.2–70.4)			(79.0–88.3)			(82.0–90.7)			(78.6–84.6)
One CCA (urine day 1)	138	72	52.2	245	171	69.8	242	173	71.5	625	416	66.6
			(43.8–60.5)			(64.0–75.5)			(65.8–77.2)			(62.9–70.3)
One CCA-L (urine day 1)	138	23	16.7	245	127	51.8	242	93	38.4	625	243	38.9
			(10.4–22.9)			(45.6–58.1)			(32.3–44.6)			(35.1–42.7)
***S. haematobium*** ** diagnosis**												
Three urine filtrations	138	0	0	245	0	0	242	29	12.0	625	29	4.6
									(07.9–16.1)			(3.0–6.3)
One urine filtration (urine day 1)	138	0	0	245	0	0	242	6	2.5	625	6	1.0
									(0.5–4.4)			(0.2–1.7)
Three Hemastix test	138	5	3.6	245	20	8.2	242	36	14.9	625	61	9.8
			(0.5–6.7)			(4.7–11.6)			(10.4–19.4)			(7.4–12.1)
One Hemastix test (urine day 1)	138	2	1.4	245	11	4.5	242	24	9.9	625	37	5.9
			(0.0–3.4)			(1.9–7.1)			(6.2–13.7)			(4.1–7.8)

Setting A = Yaoundé, Setting B = Makénéné, Setting C = Njombé.

For the overall three study settings A, B and C, the prevalence of *S. mansoni* increased from 32.8% with one Kato-Katz to 41.8% with three Kato-Katz, and 61.0% with nine Kato-Katz thick smear readings.

### CCA test results (Table 1)

Similarly, there was an increase of infection prevalence with the increase of the number of CCA tests. The prevalence of *S. mansoni* infections as determined by urine CCA were 52.2% and 62.3% in setting A, 69.8% and 83.7% in setting B, and 71.5% and 86.4% in setting C for one CCA and three CCA tests, respectively. For the overall three settings A, B and C the *S. mansoni* infection prevalence increased from 66.6% with one CCA to 81.6% with three CCA tests.

For CCA-L, the *S. mansoni* infection prevalence after a single test was 16.7% in setting A, 51.8% in setting B, 38.4% in setting C, and 38.9% in the overall three settings.

### Agreement between Kato-Katz and CCA assays

Table 2 shows the agreement between the different diagnostics approaches and the reference test, i.e. nine Kato-Katz thick smears, for the diagnosis of *S. mansoni*, stratified by study settings. There were a substantial agreement between the nine Kato-Katz thick smears and one Kato-Katz in setting A (k = 0.6), and three Kato-Katz in settings A (k = 0.7) and C (k = 0.6). The agreement was moderate with one Kato-Katz in setting B (k = 0.4) and three Kato-Katz in setting B (k = 0.5); and with one CCA and CCA-L in all three settings (k = 0.4–0.5). Finally, the agreement was fair with three CCA in all three settings (k = 0.3–0.4) and with one Kato-Katz in setting C (k = 0.4).

**Table 2:** Agreement between different techniques for the diagnosis of *S. mansoni* infections.

		Setting A				Setting B				Setting C				All settings A-B-C			
Diagnostic test	Test result	Nine Kato-Katz thick smears				Nine Kato-Katz thick smears				Nine Kato-Katz thick smears				Nine Kato-Katz thick smears			
		Positive	Negative	K*	P-value	Positive	Negative	K*	P-value	Positive	Negative	K*	P-value	Positive	Negative	K*	P-value
One Kato-Katz (stool day 1)	Positive	29	0	0.63	8.8 10^−16^	100	0	0.42	4.4 10^−16^	76	0	0.4	3.1 10^−15^	205	0	0.47	0
	Negative	21	88			76	69			79	87			176	244		
Three Kato-Katz (stool 1)	Positive	33	0	0.71	0	120	0	0.54	0	108	0	0.62	0	261	0	0.62	0
	Negative	17	88			56	69			47	87			120	244		
One CCA (urine day 1)	Positive	41	31	0.42	1.14 10^−7^	145	26	0.43	7.1 10^−12^	136	37	0.47	7.6 10^−14^	322	94	0.47	0
	Negative	9	57			31	43			19	50			59	150		
Three CCA	Positive	46	40	0.4	5.84 10^−8^	160	45	0.29	9.8 10^−7^	149	60	0.31	3.4 10^−9^	355	145	0.37	0
	Negative	4	48			16	24			6	27			26	99		
One CCA-L	Positive	23	0	0.52	3.1 10^−12^	121	6	0.49	0	86	7	0.41	3.3 10^−13^	230	13	0.5	0
	Negative	27	88			55	63			69	80			151	231		

* k<0 indicating no agreement, k = 0–0.2 indicating poor agreement, k = 0.21–0.4 indicating fair agreement, k = 0.41–0.6 indicating moderate agreement, k = 0.61–0.8 indicating substantial agreement, and k = 0.81–1 indicating almost perfect agreement.

### Sensitivity, specificity and predictive values of CCA tests

The sensitivity, specificity, positive predictive value (PPV) and negative predictive value (NPV) of the different diagnostic tests were determined and the results are summarised in Table 3. Using the nine Kato-Katz thick smears as ‘reference’ diagnostic test, the sensitivity varied from 46% to 96.1%. In all three settings, there was an increase of sensitivity with the increase from single to triplicate Kato-Katz or CCA tests. The lowest sensitivity was obtained with CCA-L in setting A (46%). However, when considering the overall three settings, the lowest sensitivity was obtained with single Kato-Katz. The triplicate CCA produced the highest sensitivity in setting C (96.1%) and for the overall three settings (93.2%), followed by the single CCA (84.5%) and the triplicate Kato-Katz (68.5%).

**Table 3:** Sensitivity, specificity, positive predictive value (PPV) and negative predictive value (NPV) of different tests for the diagnosis of *S.mansoni*.

	Setting A				Setting B				Setting C				All settings A-B-C			
	Sensitivity	Specificity	PPV	NPV	Sensitivity	Specificity	PPV	NPV	Sensitivity	Specificity	PPV	NPV	Sensitivity	Specificity	PPV	NPV
	% CI	% CI	% CI	% CI	% CI	%CI	% CI	% CI	% CI	% CI	% CI	% CI	% CI	% CI	% CI	% CI
**Nine Kato-Katz as ‘reference’ diagnostic test**																
Single Kato-Katz (stool day 1)	58	100	100	80.7	56.8	100	100	47.6	49.0	100	100	52.4	53.8	100	100	58.1
	(44.3–71.7)	/	/	(73.3–88.1)	(49.5–64.1)	/	/	(39.5–55.7)	(41.2–56.9)	/	/	44.8–60.0)	(48.8–58.8)	/	/	(53.4–62.8)
Triplicate Kato-Katz (stool day 1)	66	100	100	83.8	68.2	100	100	55.2	69.7	100	100	64.9	68.5	100	100	67
	(52.9–79.1)	/	/	(76.8–90.9)	(61.3–75.1)	/	/	(46.5–63.9)	(62.4–76.9)	/	/	(56.8–73.0)	(63.8–73.2)	/	/	(62.2–71.9)
Triplicate CCA	92	54.5	53.5	92.3	90.9	34.8	78.0	60	96.1	69.0	71.3	81.8	93.2	40.6	71	79.2
	(84.4–99.5)	(44.1–64.9)	(42.9–64.0)	(85.1–99.6)	(86.7–95.2)	(23.5–46.0)	(72.4–83.7)	(44.8–75.2)	(93.1–99.2)	(59.2–78.7)	(65.2–77.4)	(68.7–95.0)	(90.6–95.7)	(34.4–46.7)	(67.0–75.0)	(72.1–86.3)
Single CCA (urine day 1)	82	64.7	56.9	86.4	82.4	62.3	84.8	58.1	87.7	42.5	78.6	72.5	84.5	61.5	77.4	71.8
	(71.6–92.6)	(54.8–74.8)	(45.5–68.4)	(78.1–94.6)	(76.8–88.0)	(50.9–73.8)	(79.4–90.2)	(46.9–69.3)	(82.6–92.9)	(32.1–52.9)	(72.5–84.7)	(61.9–83.0)	(80.9–88.1)	(55.4–67.6)	(73.4–81.4)	(65.7–77.9)
CCA-L	46	100	100	76.5	68.8	91.3	95.3	53.4	55.5	92.0	92.5	53.7	60.4	94.7	94.7	60.5
	(32.2–59.8)	/	/	(63.8–84.3)	(61.9–75.6)	(84.7–98.0)	(91.6–99.0)	(44.4–62.4)	(47.7–63.3)	(86.2–97.7)	(87.1–97.8)	(45.7–61.7)	(55.5–65.3)	(91.9–97.5)	(91.8–97.5)	(55.6–65.4)
**Combined results as ‘reference’ diagnostic test**																
Single Kato-Katz (stool day 1)	32.2	100	100	44.0	45.2	100	100	16.6	35.3	100	100	16.3	39.	100	100	23.6
	(22.6–41.9)	/	/	(34.7–53.4)	(38.7–51.8)	/	/	(10.5–22.6)	(29.0–41.7)	/	/	(10.7–21.9)	(34.8–43.1)	/	/	(19.5–27.6)
Triplicate Kato-Katz (stool day 1)	36.7	100	100	45.7	45.3	100	100	19.2	50.2	100	100	20.1	49.7	100	100	27.2
	(26.7–46.6)	/	/	(36.2–55.2)	(47.7–60.9)	/	/	(12.3–26.1)	(43.5–56.9)	/	/	(13.4–26.9)	(45.3–53.9)	/	/	(22.6–31.8)
Single CCA (urine day 1)	80	100	100	92.3	77.4	100	100	60	80.5	100	100	81.8	79.1	100	100	79.2
	(71.7–88.3)	/	/	(85.1–99.6)	(71.9–82.9)	/	/	(44.8–75.2)	(75.2–85.8)	/	/	(68.7–95.0)	(75.6–82.6)	/	/	(72.1–86.3)
Triplicate CCA	95.6	100	100	72.7	92.8	100	100	32.4	97.2	100	100	39.1	95.1	100	100	47.4
	(91.3–99.8)	/	/	(62.0–83.5)	(89.3–96.2)	/	/	(21.8–43.1)	(95.0–99.4)	/	/	(27.6–50.6)	(93.2–96.9)	/	/	(40.6–54.1)
CCA-L	25.6	100	100	41.7	57.5	100	100	20.3	43.3	100	100	18.1	46.2	100	100	25.9
	(16.5–34.6)	/	/	(32.7–50.8)	(50.9–64.0)	/	/	(13.1–27.6)	(36.6–49.9)	/	/	(11.9–24.3)	(41.9–50.5)	/	/	(21.5–30.3)

The specificity of CCA test was higher with CCA-L, reaching 100% in setting A. Apart from the setting C, the specificity of CCA assays increased from single to triplicate tests. For the overall three settings, the specificity increased from 40.6% with triplicate CCA to 61.5% with single CCA and 94.7% with CCA-L.

There was a significant variation of PPV and NPV of CCA and CCA-L between study sites, with a higher PPV value of 100% for CCA-L in setting B and a lower value of 53.5% for triplicate CCA in setting A. The NPV varied from 53.4% for CCA-L in setting B to 92.3% for triplicate CCA in setting A (Table 3).

In addition to the reference nine Kato-Katz, the sensitivity of the different assays was assessed towards the combined results of all tests (nine Kato-Katz, triplicate CCA and single CCA-L). Similar trends of increased sensitivities with the increase of number of tests were obtained (Table 3).

### Effects of *S. mansoni* infection intensities

**Figure 1:**
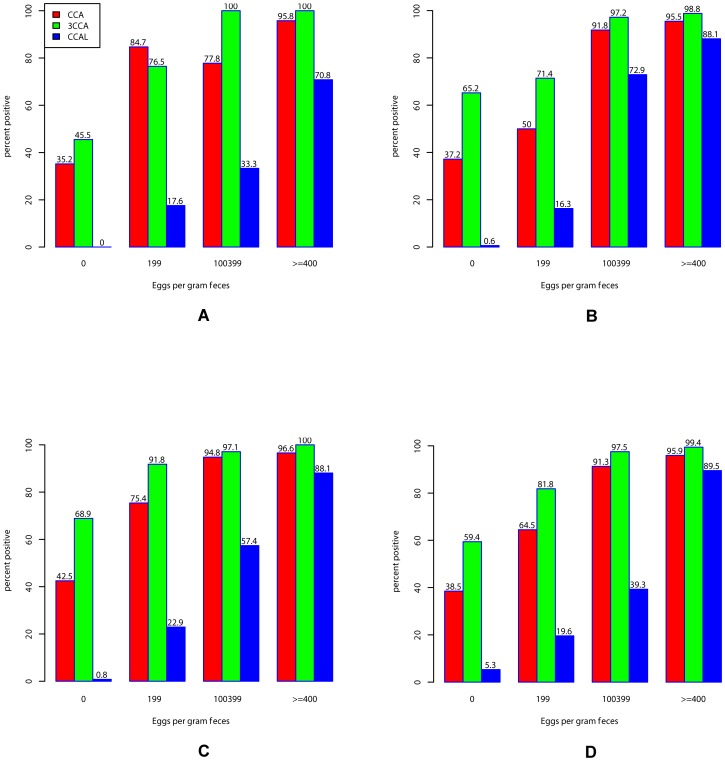
Correlation between *S. mansoni* infection intensities and CCA results. The prevalences of CCA (single CCA, triple CCA and CCA-L) are affected by the abundance of *S. mansoni* infection (stratified by intensity classes). A = setting 1, B = setting 2, C = setting 3, D = All settings A-B-C.

To determine if the CCA and CCA-L results were affected by the intensities of *S. mansoni* infections, the data were analyzed considering the different intensity classes estimated from the 9 Kato-Katz thick smear readings. The results are summarized in Table 4, and the impact of infection intensities on CCA and CCA-L results is illustrated in Figure 1. For all three assays (triplicate CCA, single CCA and single CCA-L) there was an increase of prevalence and sensitivity with the increase of *S. mansoni* infection intensities in all three settings, apart from single CCA in setting A. In moderate and heavily infected children, the prevalence of single and triplicate CCA was above 90% in all three settings. High proportions of individuals negative for nine Kato-Katz thick smears were positive for all CCA tests. Indeed, for the overall three settings, the prevalence of positive CCA among Kato-Katz negative individuals varied from 5.3% for CCA-L to 59.4% for triplicate CCA.

**Table 4:** Correlation between the prevalence of CCA test and the intensities of *S. mansoni* infections.

Diagnostic test	Intensity of *S. mansoni*	Setting A	Setting B	Setting C	All Settings A-B-C
		% positive (95% CI)	% positive (95% CI)	% positive (95% CI)	% positive (95% CI)
Three CCA	Negative	45.5 (35.1–55.9)	65.2 (54.0–76.5)	69.0 (59.2–78.7)	59.4 (53.3–65.6)
	Low	76.5 (56.3–96.6)	71.4 (58.8–84.1)	91.8 (84.9–98.7)	81.9 (75.2–88.6)
	Moderate	100	97.3 (92.1–100)	97.1 (91.6–100)	97.5 (94.2–100)
	Heavy	100	98.9 (96.7–100)	100	99.4 (98.3–100)
One CCA (urine day 1)	Negative	35.2 (25.2–45.2)	37.7 (26.2–49.1)	42.5 (32.1–52.9)	38.5 (32.4–44.6)
	Low	84.7 (42.0–87.4)	50.0 (37.0–65.0)	75.4 (64.6–86.2)	64.6(56.2–72.9)
	Moderate	77.8 (50.6–100)	91.9 (83.1–100)	94.3 (86.6–100)	91.4 (85.2–97.5)
	Heavy	95.8 (87.8–100)	95.6 (91.3–99.8)	96.6 (92.0–100)	96.0 (93.0–98.9)
One CCA-L (urine day 1)	Negative	0	08.7 (2.1–15.4)	08.0 (2.3–13.8)	5.3 (2.5–8.1)
	Low	17.6 (00–35.8)	16.3 (6.0–26.7)	23.0 (12.4–35.5)	19.7 (12.8–26.6)
	Moderate	33.3 (2.5–64.1)	73.0 (58.7–87.3)	57.1 (40.7–73.5)	39.4 (30.9–47.9)
	Heavy	70.8 (52.6–89.0)	95.6 (91.3–99.8)	88.1 (79.9–96.4)	89.6 (85.0–94.1)

### Effects of *S. haematobium* infections and micro-haematuria

In setting C, the prevalence of *S. haematobium* increased from 2.5% with one urine filtration to 12.0% with three filtrations. Similarly, the prevalence of micro-haematuria increased from 9.9% after one test to 14.9% after three tests on three consecutive days (Table 1). Interestingly, micro-haematuria was also found in settings A (3.6%) and B (8.2%), non endemic for *S. haematobium* and where all urine filtrations were negative. These 25 children with micro-haematuria were aged between 7–14 years. They were mainly girls, with only 4 boys.

A logistic regression analysis and adjustments were used to assess the correlation between CCA test categories and *S. haematobium* egg counts or microhaematuria. The results showed the absence of correlation between the CCA and CCA-L positivity and the concurrent infection with *S. haematobium*. OR = 0.9 (p = 0.10) for triple CCA, 0.9 (p = 0.16) for single CCA and 0.87 (p = 0.22) for CCA-L. Similarly, there was no significant association between the CCA or CCA-L positivity and the presence of micro-haematuria (p>0.05).

### Effects of STH infections

In order to assess the potential effects of infections with *Ascaris lumbricoides*, *Trichuris trichiura* or hookworms, the CCA and CCA-L responses was compared between individuals non infected and infected with any of these three species of soil-transmitted helminths (STH). The results show no significant difference of percentages of positive CCA and CCA-L based on STH infections status of individuals; 54.2% vs 62.1% for triple CCA, 36.1% vs 39.7% for single CCA and 6% vs 5% for CCA-L, for STH positive and negative individuals, respectively. This suggests that STH infections do not influence the urine CCA assay results.

### Day to day variation of CCA tests

Detailed analysis of the data showed a day-to-day variation of the results of CCA in 21 children: 5 in setting A, 8 in setting B and 8 in setting C. It appears that in these few 21 individuals (16 positive for Kato-Katz and 5 negative) the CCA test varied from positive to negative from one day another, and *vice versa*, even in children positive for Kato-Katz, i.e. infected with *S. mansoni*.

### Discriminating power of CCA tests: ROC curves and AUC

**Figure 2:**
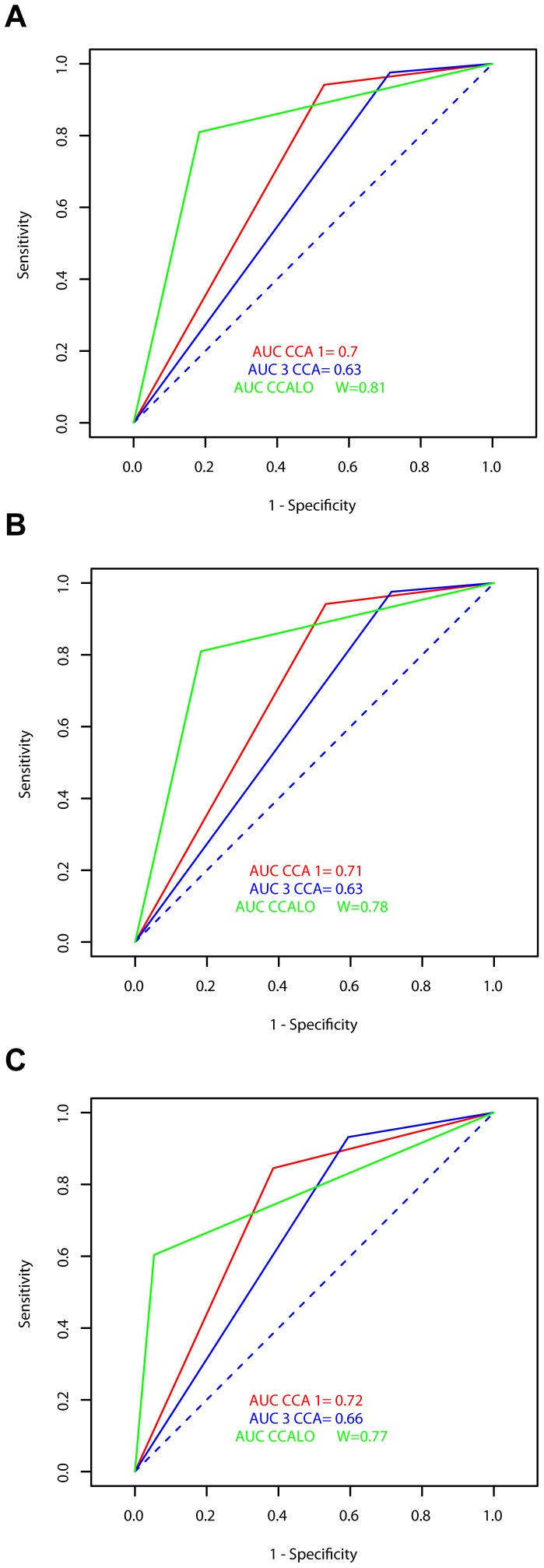
ROC curves and AUC of CCA results using Kato-Katz as reference tests. The receiver operating characteristic (ROC) curves and the area under the curve (AUC) of CCA and CCA-L are presented using either single (A), triplicate (B) and nine Kato-Katz (C) thick smears as the reference tests. The discriminating powers of single CCA and CCA-L were high in all cases.

To assess the discriminating power of CCA tests, data of all three settings were merged together. The ROC curves and AUC of the different CCA and CCA-L assays are presented in Figure 2. It appears that the discriminating powers of single CCA and CCA-L were high in all cases, as the values of AUC were >0.7. However, triplicate CCA was less discriminating, with values of AUC (0.6–0.7)<0.7.

## Discussion

The urine-CCA cassette test is a rapid antigen detecting test of active schistosome infection, more specifically *S. mansoni*. As the gastrointestinal tract of schistosome is a cul-de-sac, the parasite has to regurgitate at regular intervals the undigested particulate material as well as parasitic gut associated glycoproteins. CCA is one of the major antigens regurgitated by adult schistosomes and the major portion is later secreted in the host urine. The results of our study showed that urine CCA tests gave higher prevalences of *S. mansoni* than single and multiple Kato-Katz thick smears in all three investigated settings. This higher sensitivity of CCA is consistent with previous studies [19], [20]. However, detailed analysis of data revealed the complexity of results.

In the three epidemiological settings, the *S. mansoni* prevalence increased by about twofold when moving from single to nine Kato-Katz thick smears. This result confirms the low sensitivity of single Kato-Katz and its improvement with the increase of the number of thick smears [21]–[23]. Taking into account the fact that repeating Kato-Katz allows a better estimation of the ‘true’ prevalence of schistosome infection, we adopted a 3×3 Kato-Katz design, i.e. 3 stool samples collected per child on three consecutive days and 3 Kato-Katz readings per sample; and the cumulative results of the 9 Kato-Katz thick smear readings was used as our ‘reference’ diagnostic test. The sensitivity and specificity of the POC-CCA and CCA-L assays were determined by comparison to this reference test. The sensitivity of the normal CCA was high (>82%) in all three settings, whereas the specificity was low, ranging from 42.5% to 64.7%. On the contrary, the sensitivity of CCA-L was low (overall 60%) and the specificity was quite high, above 91% in settings B and C, and up to 100% in setting A. This showed the poor accuracy of CCA-L in detecting *S. mansoni* infections. Therefore, this assay, in its current formulation, cannot be recommended for *S. mansoni* diagnosis. Similar results were obtained by Coulibaly et al. [23]. In all three settings, and for the overall settings, a single CCA showed higher or similar high prevalence than nine Kato-Katz thick smears. This suggests that the current commercially available CCA urine cassette assay maybe an appropriate test for the diagnosis of *S. mansoni* in moderate transmission zones, as reported by previous studies in different settings [20], [24].

Detailed analysis of our data showed a strong correlation between the *S. mansoni* infection intensities and the positivity rate of CCA, as well as the intensity of CCA test bands. In all settings, CCA prevalences were above 95% and up to 100% in children with high burden of infections (infection intensity ≥400 epg), whereas they were only about 65% in light infected children (Figure 1). This high variation of the sensitivity of CCA tests with the intensity of infections is in line with previous studies [19], [20], [25], [26]. Interestingly, our studies showed no influence of STH infections on the CCA results. Furthermore, there was no cross reactivity of *S. haematobium* infections nor microhaematuria (determined by urinalysis reagent strips) on the CCA test results. Similar results were obtained in recent studies in Kenya [20] and Côte d'Ivoire [23].

Although far less than for Kato-Katz, day-to-day fluctuations of CCA results were observed in few children all three settings. This daily variation of CCA results from positive to negative and vice versa were observed in 3.4% (21/625) of children. From these 21 children with day-to-day fluctuation in urine CCA, 4 had positive Kato-Katz for all stools collected on the three consecutive days, 6 were negative for all nine Kato-Katz, and 11 exhibited day-to-day variation in Kato-Katz results. Apart from two children with moderate infection intensities, the daily fluctuation of CCA was observed mainly in children with very light infection, with an overall mean *S. mansoni* egg counts of 1.7 epg. This variation of CCA test results might be linked to the day-to-day fluctuation of schistosome CCA levels in urine of humans infected with *S. mansoni*, as previously demonstrated in several countries [27]–[29]. Nevertheless, the fact that CCA is negative in those infected children with egg excretion detected by Kato-Katz raised concerns about the accuracy of CCA for diagnosing *S. mansoni* infections. According to the manufacturer's leaflet, false CCA negative during the parasitic developing phase, usually in the first 4–8 weeks after infection. However, once adult worms start laying eggs, as detected by Kato-Katz, it becomes difficult to explain false CCA negative results without recourse to more detailed physiological examinations of the worms themselves. Further studies in the laboratory, perhaps, maybe required to address this issue.

The comparison of single CCA and triplicate CCA results showed that the sensitivity of CCA increases with repetition of tests, whereas the specificity decreases. This is confirmed by the value the value of AUC (<70%). This indicates that repeating CCA tests significantly increases the number of false positive individuals to the detriment of true positive.

In conclusion, considering the fact that urine CCA cassette produced similar prevalence as nine Kato-Katz thick smears, this assay seems an attractive tool for the diagnosis of *S. mansoni* infections. Moreover, its rapidity, easy to use, less time consuming than Kato-Katz, and the relative easiness to collect urine than stool samples are significant advantages for this test. Its field applicability for large scale screening for *S. mansoni* infections would be very useful in control programmes, especially as we are moving toward schistosomiasis elimination where feasible. However, further improvement of the assay and efforts to reduce the cost of CCA cassettes are required to enable its large scale. On the contrary, the results of the CCA-L dipstick, in its current formulation, were not satisfactory.
